# Relationship Between Lipid Profile and Body Mass Index in Patients With Polycystic Ovarian Syndrome: A Cross-Sectional Study Among Indian Women of Reproductive Age

**DOI:** 10.7759/cureus.63251

**Published:** 2024-06-26

**Authors:** Aparna Shukla, Renu Singh

**Affiliations:** 1 Centre for Advanced Research, King George's Medical University, Lucknow, IND; 2 Department of Obstetrics and Gynaecology, King George's Medical University, Lucknow, IND

**Keywords:** bmi, pcos, infertility, lipid profile, cardiovascular risk

## Abstract

Introduction

Characterized by a range of symptoms including irregular menstrual cycles, hirsutism, and infertility, polycystic ovarian syndrome (PCOS) also poses substantial metabolic challenges. Among these, dyslipidemia and obesity are particularly concerning due to their long-term implications for cardiovascular health. The present study explores the intricate relationship between lipid profile and body mass index (BMI) in patients with PCOS presenting to the Department of Obstetrics and Gynaecology, King George's Medical University, Lucknow, a tertiary care teaching hospital in Northern India.

Methods

The present work involves analysis of clinical characteristics of 230 premenopausal women between 18-45 years of age diagnosed with PCOS (according to Rotterdam Criteria). Patients with conditions such as congenital adrenal hyperplasia; Cushing’s syndrome; uncontrolled hypertension, smoking, hypogonadism; using oral contraceptives; pregnant or lactating mothers; smoking or drug addiction; psychiatric illness; and those diagnosed with androgen-secreting tumors were excluded. For each participant, data was collected pertaining to demographics (age, marital status), clinical presentation, height, and weight (for BMI calculation), and total lipid profile comprising of total cholesterol, triglycerides, high-density lipoprotein cholesterol (HDL-C), low-density lipoprotein cholesterol (LDL-C), and very low-density lipoprotein cholesterol (VLDL-C). The patients were stratified according to their BMI as per the WHO Asian classification, and Pearson’s correlation coefficient was calculated to see the correlation between lipid profile and BMI.

Results

The final analysis was done on 228 patients, with a mean age of 25.22 (±4.82) years, and 45.6% were currently married. Among the participants, 28.1% of the participants were overweight, and 42.5% of them classified as obese, while the remaining 29.4% had a BMI in the normal range. The mean (SD) total cholesterol was 172.26 (49.55) mg/dl, and 65 patients (28.5%) had elevated serum cholesterol (≥200 mg/dl). Triglycerides were raised (≥ 150 mg/dl) in a majority of the study participants (n=160, 70.2%). While none of the patients had high VLDL-C, LDL-C was elevated in 52 patients (22.8%). HDL-C levels were lower than the threshold value of 50 mg/dl in 152 participants (66.7%). On performing correlation analysis, a significant negative correlation was noted between HDL-C and BMI in the entire study cohort (r=-0.342, p=0.003). Overweight patients exhibited a statistically significant positive correlation between triglycerides and BMI (r=2.628, p=0.002). Participants with BMI in the overweight range demonstrated statistically significant correlations with HDL-C (r=-0.497, p=0.017) and triglycerides (r=2.628, p=0.002); and BMI of obese patients significantly correlated with total cholesterol (r=0.301, p=0.037) and triglycerides (r=0.146, p=0.028).

Conclusion

The findings of the study underscore the multifaceted nature of PCOS, affecting reproductive health as well as metabolic components. The significant correlations between BMI and lipid parameters, specifically, HDL-C, triglycerides, and total cholesterol, highlight the importance of weight management in reducing cardiovascular risks in women with PCOS.

## Introduction

Polycystic ovarian syndrome (PCOS) is a common endocrine disorder that affects around 5-18% of women of reproductive age, and more than 50% of women either remain undiagnosed or have delayed diagnosis [1]. The prevalence of PCOS seems to be increasing worldwide due to the westernization of lifestyle. A recent systematic review and meta-analysis indicated that approximately 10% of Indian women are affected by PCOS when evaluated using the Rotterdam and Androgen Excess Society (AES) criteria [2]. Characterized by a range of symptoms including irregular menstrual cycles, hirsutism, and infertility, PCOS also poses substantial metabolic challenges. Among these, dyslipidemia and obesity are particularly concerning due to their long-term implications for cardiovascular health [3].

Prior evidence establishes dyslipidemia as a frequent metabolic abnormality in young adult women with PCOS [4], with prevalence reported to be as high as 70% [5]. Dyslipidemia in PCOS is consistent with that observed in an insulin-resistant state [6]. Obesity and insulin resistance remain the cornerstones of a vicious cycle, each exacerbating the other, and significantly contributing to the development of metabolic syndrome in PCOS patients [7]. Insulin resistance, a common feature of PCOS, not only promotes weight gain but also alters lipid metabolism, leading to abnormal lipid profiles [7,8]. Conversely, increased adiposity further impairs insulin sensitivity, perpetuating the cycle of metabolic dysfunction [7,9].

The present study explores the intricate relationship between lipid profile and body mass index (BMI) in patients with PCOS presenting to the Department of Obstetrics and Gynaecology, King George's Medical University, Lucknow, a tertiary care teaching hospital in Northern India. By examining how BMI influences lipid levels and vice versa, we aim to shed light on the metabolic intricacies of PCOS and highlight the importance of early intervention and tailored treatment strategies in managing these interrelated conditions. Understanding these connections is crucial for improving clinical outcomes and enhancing the quality of life for women affected by this multifaceted syndrome.

## Materials and methods

Study design and participants

The present work involves analysis of clinical characteristics of premenopausal women between 18-45 years of age diagnosed with PCOS (according to Rotterdam Criteria) and attending the OPD of the Department of Obstetrics and Gynaecology, King George’s Medical University (KGMU), Lucknow for treatment. Data collection was initiated after approval from the Institutional Ethics Committee, KGMU, Lucknow (No. 208/Ethics/2019 dated 13-3-2019). Patients not willing to participate and did not give informed consent in writing for the study; those with conditions such as congenital adrenal hyperplasia, Cushing’s syndrome; acute or chronic medical illness (including hepatic, cardiac or renal insufficiency, COPD, gastrointestinal disorders); uncontrolled hypertension, smoking, and hypogonadism; using oral contraceptives; pregnant or lactating mothers; smoking or drug addiction; psychiatric illness; and those diagnosed with androgen-secreting tumors were excluded.

Study procedure

A total of 230 patients meeting the eligibility criteria were enrolled in the study, after explaining the purpose and procedure of the study and obtaining written informed consent. Detailed demographic data (age and marital status) and clinical presentation (chief complaints, duration of symptoms, associated features of hyperandrogenism) were recorded at the time of patient enrolment. Anthropometric measurements included measurement of height and weight according to standard protocols. Venous samples were collected after overnight fasting of 8-12 hours for estimation of various blood parameters which included serum lipids comprising of total cholesterol, triglycerides, high-density lipoprotein cholesterol (HDL-C), low-density lipoprotein cholesterol (LDL-C), and very low-density lipoprotein cholesterol (VLDL-C).

Total cholesterol and triglyceride (TG) levels were determined in the serum by a commercially available kit. HDL-C was measured by using the direct HDL method. LDL-C and VLDL-C were calculated according to the formula of Friedewald et al., given as LDL-C = cholesterol − [HDL-C + (TG/5)], where the last term, TG/5, is an estimate of VLDL-C [10]. Individual parameters were considered elevated when the values exceeded the threshold values of 200 mg/dl for total cholesterol, 150 mg/dl for triglycerides, 130 mg/dl for LDL-C, 40 mg/dl for VLDL-C and below 50 mg/dl for HDL-C.

Data analysis

The data entry was done in Microsoft Excel 2016 (Microsoft Office 2016 package) (Microsoft Corporation, Redmond, WA) and analysis was done using the statistical software SPSS for Windows, Version 16.0 (SPSS Inc., Chicago, IL). Descriptive statistics included expression of continuous variables as mean ± standard deviation (SD), and categorical variables as relative frequency and percentage. For the present study, the patients were stratified according to their BMI as per WHO classification: normal - 18.5-24.9 kg/m^2^, overweight - 25-29.9 kg/m^2^, and obese - ≥30 kg/m^2^. Pearson’s correlation coefficient was calculated to see the correlation between lipid profile and BMI. A p-value <0.05 was considered as statistically significant.

## Results

A total of 230 participants were initially recruited for the study, of whom complete baseline data of 228 patients were available. And were thus included in the final analysis. The majority of the study participants (n=103, 45.2%) belonged to the age group of 21-25 years, followed by 78 patients aged between 26-30 years (34.2%). There were 11 patients with PCOS (4.8%) aged more than 35 years. The mean (±SD) age was 25.22 (±4.82) years. Of the study participants, 45.6% (n=104) were married, of whom, 40 reported infertile (failure to conceive after 12 months or more of regular unprotected sexual intercourse) comprising 38.4% of the married women. Of the participants, 28.1% were overweight (n=64), and 42.5% of them classified as obese (n=97), while the remaining 29.4% had BMI in the normal range (n=67) (Table 1).

**Table 1:** Baseline demographic and anthropometric characteristics of the study participants (n=228)

Demographic characteristics	Categories	Frequency	Percentage
Age (years)	18-20	24	10.5
	21-25	103	45.2
	26-30	78	34.2
	31-35	12	5.3
	>35	11	4.8
Marital status	Married	104	45.6
	Unmarried	124	54.4
BMI	Normal	67	29.4
	Overweight	64	28.1
	Obese	97	42.5

The most frequently reported complaint included oligomenorrhea (n=143, 62.7%) and irregularity of menstrual cycle (n=46, 20.2%). The mean (±SD) duration of complaints was 15.3 (±8.2) months. More than half of the participants were hypothyroid (n=129, 56.6%). The most common manifestation of hyperandrogenism among the study participants was reported to be acne (n=78, 34.2%), closely followed by irritable bowel syndrome (n=75, 32.9%) (Table 2).

**Table 2:** Hyperandrogenic features among the study participants (n=228)

Hyperandrogenic features	Frequency	Percentage
Acne	78	34.2
Temporal balding	17	7.5
Hyperpigmentation	17	7.5
Galactorrhea	20	8.8
Irritable bowel syndrome	75	32.9

The descriptive statistics of the serum lipid parameters are presented in Table 3. The mean (SD) total cholesterol was 172.26 (49.55) mg/dl, and 65 patients (28.5%) had elevated serum cholesterol (≥200 mg/dl). Triglycerides were raised (≥ 150 mg/dl) in a majority of the study participants (n=160, 70.2%). While none of the patients had high VLDL-C, LDL-C was elevated in 52 patients (22.8%). HDL-C levels were lower than the threshold value of 50 mg/dl in 152 participants (66.7%).

**Table 3:** Descriptive statistics of the serum lipid parameters among the study participants (n=228) HDL-C: high-density lipoprotein cholesterol; LDL-C: low-density lipoprotein cholesterol; VLDL-C: very low-density lipoprotein cholesterol

Parameters	Cholesterol (mg/dl)	Triglycerides (mg/dl)	HDL-C (mg/dl)	LDL-C (mg/dl)	VLDL-C (mg/dl)
Mean	172.26	177.28	46.57	90.23	35.45
Standard deviation	49.55	60.42	9.19	45.94	12.08
Median	166.50	170.00	47.00	81.26	34.00
1^st^ quartile	128.00	135.00	39.00	53.75	27.00
3^rd^ quartile	206.50	210.00	53.75	123.55	42.00
Minimum	92.00	59.00	26.50	4.00	11.80
Maximum	310.00	340.00	68.00	211.80	68.00

On performing correlation analysis, a significant negative correlation was noted between HDL-C and BMI in the entire study cohort (r=-0.342, p=0.003). On stratified analysis, HDL-C was found to have a negative correlation with patients in individual BMI classes, with the strongest association observed between BMI and HDL-C among overweight patients (r=-0.497, p=0.017). Besides HDL-C, participants in this group also exhibited a statistically significant positive correlation between triglycerides and BMI (r=2.628, p=0.002). Participants with BMI in the obese range demonstrated statistically significant correlations with total cholesterol (r=0.301, p=0.037) and triglycerides (r=0.146, p=0.028) (Table 4).

**Table 4:** Correlation between BMI categories and serum lipid profile among the study participants HDL-C: high-density lipoprotein cholesterol; LDL-C: low-density lipoprotein cholesterol; VLDL-C: very low-density lipoprotein cholesterol * indicates statistically significant at p<0.05

Lipid parameter	Total (n=228)		Normal BMI (n=67)		Overweight (n=64)		Obese (n=97)	
	r	p-value	r	p-value	r	p-value	r	p-value
Total cholesterol	0.248	0.915	-0.068	0.583	0.116	0.361	0.301	0.037*
Triglycerides	1.865	0.053	0.507	0.646	2.628	0.002*	0.146	0.028*
HDL-C	-0.342	0.003*	-0.216	0.033*	-0.497	0.017*	-0.329	0.001*
LDL-C	0.108	0.906	0.183	0.502	0.129	0.822	0.108	0.937
VLDL-C	0.058	0.385	0.074	0.668	0.326	0.900	0.173	0.089

## Discussion

This study presents a detailed analysis of a cohort of women in the reproductive age group suffering from PCOS, revealing significant insights into their demographic, clinical, and metabolic characteristics and exploring the degree of correlation between the different blood lipid components and BMI. Almost half of our study participants were in their early twenties and a near similar figure were married. The young, predominantly unmarried, women presented primarily with complaints of irregular or delayed periods. In married women, PCOS often manifests as infertility, highlighting the reproductive challenges associated with PCOS. We also observed varied clinical manifestations of hyperandrogenism, with acne and IBS being the commonest of them. These findings illustrate the broad spectrum of PCOS manifestations beyond reproductive health, typically resulting from the complex pathophysiological disruption of the normal hypothalamo-pituitary-ovarian axis, compounded by insulin resistance, which leads to high levels of free circulating androgens in the blood [11,12].

A considerable portion of the participants were either overweight (28.1%) or obese (42.5%). This high prevalence of elevated BMI among the participants highlights the strong association between PCOS and obesity. Evidence suggests that PCOS is linked to impairments in insulin sensitivity and secretion, which are worsened by the presence of obesity [13]. Dyslipidemia was a frequent finding among our patients with PCOS, specifically, elevated triglycerides and reduced HDL-C were present in more than two-thirds of the study participants. A similar profile of lipid components has been reported in prior studies on PCOS women [14-16]. PCOS-associated dyslipidemia indicates a heightened risk of cardiovascular diseases in such women, underscoring the importance of cardiovascular risk assessment and management in this population.

Correlation analysis provided further insights into the relationship between BMI and lipid profiles. HDL-C levels, responsible for atherogenic dyslipidemia, were found to have a consistent negative correlation with BMI. This negative correlation was particularly strong among overweight individuals, highlighting that those who are overweight may face a greater risk of cardiovascular issues compared to those with normal weight or obesity. Additionally, a significant positive correlation was found between triglycerides and overweight status, indicating that overweight individuals with PCOS are more likely to have elevated triglycerides. Similarly, positive correlations were observed between total cholesterol and BMI as well as triglycerides and BMI in obese participants.

Similar to our observations, Kiranmayee et al. also reported a negative correlation of HDL-C in normal BMI, overweight, and obese women with PCOS, while triglycerides were reported to have a positive correlation with BMI among overweight patients [14]. Although we did not find any association between LDL-C and BMI, studies by Wild et al. and Saghafi-Asl et al. reported a positive correlation between BMI and total cholesterol and LDL-C [16,17]. The study by Rocha et al. demonstrated that BMI significantly affected HDL levels in women with PCOS, which is consistent with our findings [18]. On the contrary, a study on young Korean women with PCOS reported a significant increase in the prevalence of dyslipidemia, irrespective of their BMI status [19]. However, studies conducted on Indian PCOS patients reported a high prevalence of obesity and associated lipid abnormalities [20,21].

Dyslipidemia is a common metabolic derangement observed in PCOS. Among our participants, HDL-C, triglycerides, and total cholesterol were the most important lipid components that were correlated with BMI. These findings emphasize the metabolic challenges associated with higher BMI in PCOS patients and the need for effective management strategies to address these issues. The strength of the present study is the inclusion of a large sample size but is limited by the non-consideration of other anthropometric parameters such as waist circumference and waist-hip ratio. Also, we did not stratify our analyses according to the different phenotypic variations in PCOS.

## Conclusions

In conclusion, the findings of the study underscore the multifaceted nature of polycystic ovarian syndrome (PCOS), affecting reproductive health as well as metabolic components. The significant correlations between BMI and lipid parameters, specifically, high-density lipoprotein cholesterol, triglycerides, and total cholesterol highlight the importance of weight management in reducing cardiovascular risks in women with PCOS. These insights are crucial for tailoring comprehensive treatment approaches that address both the reproductive and metabolic aspects of PCOS, therefore, improving the overall health and quality of life for these patients.
